# The Clinical Trials of Mesenchymal Stromal Cells Therapy

**DOI:** 10.1155/2021/1634782

**Published:** 2021-11-03

**Authors:** Mohammad Reza Kouchakian, Neda Baghban, Seyedeh Farzaneh Moniri, Mandana Baghban, Shabnam Bakhshalizadeh, Vahid Najafzadeh, Zahra Safaei, Safoura Izanlou, Arezoo Khoradmehr, Iraj Nabipour, Reza Shirazi, Amin Tamadon

**Affiliations:** ^1^Department of Anatomical Sciences, School of Medicine, Iran University of Medical Sciences, Tehran, Iran; ^2^The Persian Gulf Marine Biotechnology Research Center, The Persian Gulf Biomedical Sciences Research Institute, Bushehr University of Medical Sciences, Bushehr, Iran; ^3^Department of Anatomical Sciences, School of Medicine, Tehran University of Medical Sciences, Tehran, Iran; ^4^Department of Obstetrics and Gynecology, School of Medicine, Shiraz University of Medical Sciences, Shiraz, Iran; ^5^Reproductive Development, Murdoch Children's Research Institute, Melbourne, Victoria, Australia; ^6^Department of Paediatrics, University of Melbourne, Melbourne, Victoria, Australia; ^7^Department of Veterinary and Animal Sciences, Anatomy & Biochemistry Section, University of Copenhagen, Copenhagen, Denmark; ^8^Department of Obstetrics and Gynecology, School of Medicine, Amir Al Mo'menin Hospital, Amir Al Mo'menin IVF Center, Arak University of Medical Sciences, Arak, Iran; ^9^Department of Nursing, School of Nursing, Larestan University of Medical Sciences, Larestan, Iran; ^10^Department of Anatomy, School of Medical Sciences, Medicine & Health, UNSW Sydney, Sydney, Australia

## Abstract

Mesenchymal stromal cells (MSCs) are a heterogeneous population of adult stem cells, which are multipotent and possess the ability to differentiate/transdifferentiate into mesodermal and nonmesodermal cell lineages. MSCs display broad immunomodulatory properties since they are capable of secreting growth factors and chemotactic cytokines. Safety, accessibility, and isolation from patients without ethical concern make MSCs valuable sources for cell therapy approaches in autoimmune, inflammatory, and degenerative diseases. Many studies have been conducted on the application of MSCs as a new therapy, but it seems that a low percentage of them is related to clinical trials, especially completed clinical trials. Considering the importance of clinical trials to develop this type of therapy as a new treatment, the current paper is aimed at describing characteristics of MSCs and reviewing relevant clinical studies registered on the NIH database during 2016-2020 to discuss recent advances on MSC-based therapeutic approaches being used in different diseases.

## 1. Introduction

In general, stem cells refer to a population of undifferentiated cells that are potent for proliferation, differentiation, and self-renewal [1]. With regard to potency, stem cells belong to one of four types including unipotent, multipotent, pluripotent, and totipotent cells [2]. Stem cells are defined as unipotent if they maintain the ability to just self-renew and can only differentiate into cell types of a single tissue layer. Stem cells are defined as multipotent if they differentiate into several different cell types within a single germ layer. If they differentiate into cell types from all three germ layers, namely, ectoderm, mesoderm, endoderm, and functional gametes, they are called pluripotent stem cells [3]. Totipotent stem cells can form all cell types of the adult organism as well as extraembryonic tissues [4, 5]. Based on the origin of the tissue, stem cells are also categorized to embryonic and adult. A new type of stem cells called induced pluripotent stem cells (iPSCs) has been introduced in recent years [6]. Embryonic stem cells (ESCs), which are derived from the inner cell mass of the preimplantation blastocysts are defined as pluripotent stem cells [7, 8]. Adult stem cells are present in adult tissues and replenish senescent cells and subsequently regenerate damaged tissues. These cells, including mainly hematopoietic, neural crest-derived, and mesenchymal stromal/stem cells (MSCs), are also known as multipotent stem cells [9–12].

The aforementioned features of stem cells extensively have attracted attention of experts in stem cell biology, developmental biology, biomaterial sciences, tissue engineering, and other relevant fields for restoring damaged cells and tissues to a condition as close to its normal structure and function as possible. In other words, stem cells have developed a new and surprising scenario in regenerative medicine. Nowadays, stem cell therapy not only stands at the forefront of tissue engineering and regenerative medicine but also is increasingly developed in other medical fields such as gene and drug delivery systems [13, 14]. According to the U. S. National Library of Medicine (https://clinicaltrials.gov), a total number of 6205 clinical trials on stem cell therapy worldwide were registered till 5/18/2021. Between them, 1240 of which are related to MSC therapy.

ESCs and MSCs have a number of advantages and disadvantages in their use [15–17]. The reason for this greater tendency to MSCs than other stem cell types can be understood by comparing the advantages and disadvantages of them. Strauer and Kornowski note ESCs as highly expandable and pluripotent but limited by risk of rejection, difficult isolation, risk of malignancy, and ethical objection. They contrast that MSCs are easily obtained, expanded, compatible, and are socially acceptable [15].

Following the discovery of novel treatments for diseases in laboratory and animal models, clinical trials are essential to find treatments that work properly in humans. As abovementioned, the interest of scientists to the MSC-based therapy has increased in recent years. Therefore, the current review paper is aimed at describing characteristics of MSCs and the recent advances on MSC-based therapy.

## 2. Method

This review is aimed at answering the questions “Which characteristics of MSC has attracted the attention of scientists to apply it as a therapy?”, “How many clinical trials have been reported on the NIH database over a 5-year period of 2016-2020 and how many of them are related to completed and active trials?”, and “For which diseases have no MSC-based therapy clinical trials been reported?”. The clinical trials of MSC therapy were searched through the U. S. National Library of Medicine (https://clinicaltrials.gov) using keywords of “mesenchymal + therapy.” The period of trials was limited to 2016-2020. The result of the search showed 1240 trials. The criteria for selecting articles were their status. The completed, active, and recruiting trials were selected. Accordingly, 290 of which were selected to summarize their available data in Tables 1–3. Data summarized in Table 4 were obtained by searching key words of “mesenchymal+ therapy” and omitting diseases listed in Tables 1–3 through the PubMed database. The purpose of presenting Table 4 was to find conditions that have been treated in preclinical experiments with MSCs but are yet to be translated into clinical trials.

## 3. MSCs

Friedenstein et al. first described MSCs as a population of fibroblast-like cells in the bone marrow [18]. According to the *International Society for Cellular Therapy* (ISCT) definition, to be classified as MSCs, the cells must satisfy four minimal criteria: (i) specific surface antigen expression (>95%) including CD73, CD90, CD105, CD44, CD71, Stro-1, CD106, CD166, CD29, and ICAM-1, (ii) do not express hematopoietic markers (CD45, CD34, CD14, and CD11), endothelial (CD31), and costimulatory markers (CD80, CD86), (iii) adherence to the plastic plate surface, and (iv) capability to differentiate into osteogenic, adipogenic, and chondrogenic lineages [19].

Some studies have demonstrated that MSCs are able to transdifferentiate into nonmesenchymal cells (hepatic, renal, cardiac, neural, and Schwann cells) [20–25]. These cells express pluripotency-associated factors including OCT-4, SOX-2, and NANOG [26]. However, their expression depends on the type of MSCs and their niche [27]. The niche of MSCs is the subject of much debate and has not been fully understood yet. Nonetheless, three factors may play a critical role in the residing of MSCs: (i) expression of the receptors and adhesion molecules in MSCs [28], (ii) interaction with endothelial cells [29], and (iii) expression of signalling molecules from injured tissue [30]. However, factors such as delivery method, the age of MSCs, passage number, the population of MSCs, and the source and culture condition of MSCs can alter their residing efficacy [31].

## 4. Cell Sources of MSCs

**Figure 1:**
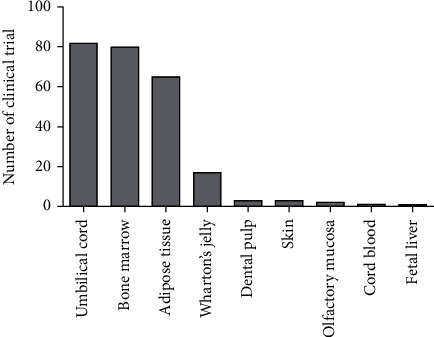
Frequency of mesenchymal stromal/stem cell (MSC) source contributions in clinical trials on MSC therapy during 2016-2020 (U. S. National Library of Medicine).

MSCs can be easily isolated from several tissues and can be effectively cultured *in vitro*. As the major sources of MSCs in clinic trials are umbilical cord, bone marrow, and adipose tissues, MSCs derived from these tissues are discussed below (Figure 1). Adult bone marrow-derived MSCs (BM-MSCs) comprise only 0.01% to 0.001% of the bone marrow cell population and can be isolated from osseous biopsies [32, 33]. BM-MSCs are multipotent and capable of differentiating into mesodermal, ectodermal, and endodermal cell lineages. Previous studies have reported that BM-MSCs are a potential source of stem cells with capability in differentiation into male germ cells [34]. It is also shown that these possess high proliferation rate and their immunomodulatory properties act through paracrine mechanisms [35–39]. Accordingly, BM-MSCs can be useful for solving some genetic and immunological problems as well as repairing damaged tissues [40]. Moreover, it has been proved that BM-MSCs secrete cytokines and growth factors and can facilitate engraftment in organs [41–43]. Adipose tissues can be considered as a suitable source for MSCs, as its harvesting is relatively noninvasive. Adipose tissue-derived stem cells (AT-MSCs) can be extracted by liposuction and isolated from the stromal vascular fraction of homogenized adipose tissues [44, 45]. These cells were first identified as MSCs by Zuk and colleagues in 2001 [46]. AT-MSCs can be isolated in high numbers, because of their abundance in the human body [47]. They express MSC markers (CD90, CD44, CD29, CD105, CD13, CD34, CD73, CD166, CD10, CD49e, and CD59) while hematopoietic and endothelial markers (CD31, CD45, CD14, CD11b, CD34, CD19, CD56, and CD146) are downregulated in this cell population [48]. More genetic and morphologic stability in long-term cultures, higher proliferation, and other characteristics give AT-MSCs a distinct advantage over BM-MSCs [49, 50]. However, their proliferation rate depends on various factors such as donor's age, fat tissue type (white or brown), location of the harvest (subcutaneous or visceral), culture conditions, cell culture density, and media formulation [51]. The human *umbilical cord* is a conduit between the developing embryo/fetus and the placenta. It contains two arteries and a vein surrounded by mucosa connective tissue called Wharton's jelly [52]. The *umbilical cord* has five various compartments including amniotic epithelium membrane, cord lining, intervascular Wharton's jelly, and perivascular and mixed cord. There is also a population of stem cells called umbilical cord-derived MSCs (UC-MSCs) that can be isolated from the *umbilical cord*. Human UC-MSCs draw attention since they are derived from a noncontroversial source, and there are no ethical concerns in harvesting and using them for treatment purposes. Human UC-MSCs are more potent possessing more proliferation potential and differentiation capacity compared to adult tissue-derived MSCs. MSCs population in Wharton's jelly is higher than the other parts of the umbilical cord [53]. In 1991, McElreavey et al. [54] isolated fibroblast-like cells from Wharton's jelly of the human umbilical cord for the first time. Wharton's jelly-derived MSCs (WJ-MSCs) express high level of MSC markers as well as some pluripotency markers [55].

## 5. Therapeutic Application of MSCs

Different types of MSC-based therapy have been studied and discussed for treating of a wide range of diseases such as graft-versus-host-disease [56–58], Crohn's disease [59, 60], type 1 diabetes [61, 62], multiple sclerosis (MS) [63, 64], lupus [65, 66], cardiovascular diseases [67, 68], liver disorders [69], respiratory disorders [70, 71], spinal cord injury [72, 73], kidney failure [74, 75], skin diseases [76, 77], Alzheimer's disease [78], and Parkinson disease [79].

## 6. Therapeutic Mechanisms of MSCs

Diverse therapeutic mechanisms have been suggested for MSCs. Exploring these mechanisms are essential to help scientist to select the suitable dosage, administration route, and best engraftment time of MSCs [85]. Several mechanisms can be involved in the therapeutic effects of MSCs on a specific disease, as described below.

The therapeutic potential of MSCs can be attributed to their secretory and immunomodulatory properties. Immunomodulatory responses are dependent on the cell-to-cell interaction mechanisms and releasing secretory factors [80, 81]. MSCs secrete a wide range of bioactive molecules including growth and antiapoptotic factors including VEGF, HGF, IGF-1, TGF-*β*, bFGF, and stanniocalcin-1 [82]. MSC-derived secretory factors induce cell proliferation and angiogenesis and limit the injury site. When the tissue is injured, molecules such as IL-1, IL-2, IL-12, TNF-*α*, and INF-*γ* produce inflammatory responses at the injury site [83]. This response prevents the regenerating process by progenitor stem cells [83]. The secretion of PGE-2, iNOS, iDO, HLA-G5, and LIF from MSCs leads to reduced inflammation and subsequent regulation of immune system cells' function [84].

Homing is a feature of MSCs referring to their tendency to home to injured tissues. This ability in MSCs, which was first identified by Saito et al. [86], is effective in treating diseases. Moreover, homing of MSCs to damaged tissues following transplantation suggests them as very promising drug carriers. MSC homing can be affected by several factors including the transplantation time and quantity, pretreatment, the method of culture, and the transplantation approach of MSCs [87, 88].

Another possible therapeutic mechanism is the differentiation of MSCs. As above-mentioned, these cells have the ability to differentiate into different cells such as adipocytes, chondrocytes, osteoblasts, myoblasts, and neuron-like cells. This ability has resulted in successful application of MSCs in tissue and scaffold engineering [89–91].

Producing trophic factor is another mechanism, as MSCs have ability to play a role as a pool of trophic factor. Following the homing MSCs in injured areas, local stimuli simulate MSCs to secrete growth factors, which have a role in tissue regeneration, angiogenesis, and preventing cell apoptosis [92–94].

## 7. Approaches for Applying MSCs

In general, eight administration methods have been proposed for applying MSCs. These methods include intravenous injection (IV) [95, 96], intra-arterial injection (IA) [97, 98], intrathecal injection (IT) [99, 100], intracardiac injection (IC) [101], intra-articular injection (IAT) [102, 103], intramuscular injection (IM) [104], intraosseous injection (IO) [105], and implant for cells incorporated into a matrix or an implanted device [106, 107]. According to the study on the clinical trials of MSCs over 2014-2018, the most common administration method used in clinical trials was IV. The next common methods were IT, IAT, IC, IM, and IO in order of applications [107].

## 8. Clinical Use of MSCs

**Figure 2:**
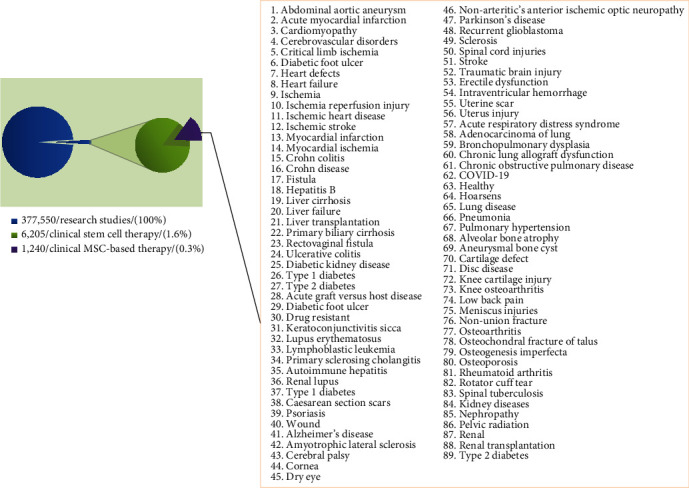
An overview on statics relating to MSC-based therapy clinical trials obtained from the U.S. National Library of Medicine.

Although MSCs offer remarkable potentials, which made them a favorable candidate for treating a large number of diseases, an overview on statics obtained from the U. S. National Library of Medicine shows that 6205 out of 377,550 research studies are related to clinical stem cell therapy (1.6%) and only 1240 of which are related to MSC-based therapy (0.3%) (Figure 2). Still, there are several concerns regarding the cell dosage and the proper administration route and timing [108–110] that limit the use of MSCs in clinical practice.

**Figure 3:**
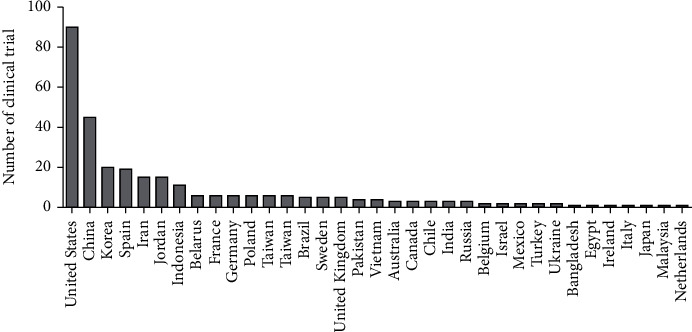
Frequency of country contributions in clinical trials on mesenchymal stromal/stem cell-based therapy during 2016-2020 (U. S. National Library of Medicine).

Some completed clinical trials in this field (all phases except early phase 1 and not applicable) over 2016-2020 have been listed in Table 1. Tables 2 and 3 show active recruiting clinical trials on MSC therapy during 2016-2020 (U. S. National Library of Medicine). According to this table, the potential of MSCs has been studied in treating numerous diseases including myocardial infarction, diabetes, spinal cord injury, and systemic lupus. Taking an overall view on these three tables, it has been found completed or active clinical tr ials were mostly related to the nervous system diseases while recruiting clinical trials were mostly related to respiratory diseases. In general, respiratory diseases have been mostly attracted researchers worldwide because of the pandemic COVID-19. In addition, the United States is ranked the first in terms of clinical trials on MSC therapy (Figure 3). Five of these studies (NCT01909154, NCT03473301, NCT02013674, NCT02958267, and NCT02509156) whose results were available have been reviewed in terms of morality rate, adverse effects, and successfulness.

NCT01909154 conducted on 12 participants to examine the safety and the impact of the local administration of autologous BM-MSCs in damaged nervous tissue. No mortality was reported in this trial. Adverse effect of urinary tract infection (12/12 or 100%), general pain and back pain (4/12 or 33.33%), myalgia and hyperthermia (3/12 or 25.00%), nasopharyngitis, nausea, muscle contracture, and headache (2/12 or 16.67%), and iron deficiency anemia, diarrhea, saline extravasation, local edema, perineal abscess, infectious mononucleosis, subcutaneous seroma, high level of cholesterol in blood, high level of alkaline phosphatase in blood, thoracic pain, intercostal neuralgia, anxiety, urinary discomfort, pressure ulcer, hemorrhoidectomy, hypertension, hypotension, and orthostatic hypotension (1/12 or 8.33%) were reported. Applying this therapy on 52 patients with spinal cord injury (SCI) showed that administration of BMSCs is safe and may increase the life quality of patients suffering from SCI [111].

NCT03473301 (A Study of UCB and MSCs in Children With CP) was conducted on a total number of 91 participants in three groups of allogeneic umbilical cord blood (AlloCB, *n* = 31), cord tissue MSCs (MSC, *n* = 29), and natural history (*n* = 31). The mortality rate was reported zero in this trial. Adverse effect including gastritis, bronchitis viral, respiratory syncytial virus, rhinovirus infection, dehydration, hypoacusis, constipation, diarrhoea, vomiting, fatigue, anaphylactic reaction, influenza, pneumonia, upper respiratory tract infection, infusion related reaction, arthropod bite, tooth avulsion, dehydration, facial paresis, partial seizures, strabismus correction, dental operation, orchidopexy, suture insertion, and tongue tie operation (1/31 or 3.23%), seizure, pyrexia, otitis media, hand-foot-and-mouth disease, and fall, laboratory test abnormal and pyrexia (2/31 or 6.45%), and surgery (3/31 or 9.68%) were observed in the AlloCB group. Upper respiratory tract infection (8/29 or 27.59%), infusion-related reaction, and rash, (4/29 or 13.79%), pyrexia (3/29 or 10.34%), otitis media, rash maculopapular, urticaria, and hospitalisation (2/29 or 6.90%), and tonsillitis, drug hypersensitivity, varicella, fall, disturbance in attention, insomnia, henoch-Schonlein purpura, orchidopexy, adenoidectomy, sleep disorder, anaemia, influenza-like illness, infusion site rash, injection site reaction, and drug hypersensitivity (1/29 or 3.45%) were reported in the MSC group. Seizure (5/31 or 16.13%), strabismus correction, hospitalization, and pyrexia (2/31 or 6.45%), and respiratory tract infection viral, otitis media, fall, fracture, adenotonsillectomy, surgery, thrombocytopenia, bradycardia, gastroesophageal reflux disease, bronchitis, and enterocolitis infectious (1/31 or 3.23%) were observed in the natural history group. Hospitalization (2/27 or 7.41%) and bronchitis, enterocolitis infectious, cough, rash, seizure, respiratory failure, cyclic vomiting syndrome, toothache, and pyrexia (1/27 or 3.70%) were observed in AlloCB after natural history. No article has been published using the results of this trial to discuss about the success of this therapy.

The clinical trial NCT02013674 was a phase II study for gaining additional safety and efficacy assessments among two-dose levels previously studied in a phase I setting. Participants included 30 patients suffering from chronic ischemic left ventricular dysfunction secondary to MI scheduled to undergo cardiac catheterization. Two groups of 20 million allogeneic hMSCs (group1, *n* = 15) and 100 million allogeneic hMSCs (group 2, *n* = 15) were designed for this trial. The mortality rate was reported 0.00% (0 l15) and 13.33% (1/15) for group 1 and group 2, respectively. Cardiac failure congestive, cardiac failure (6/15 or 60.00%), hematoma, hypotension (2/15 or 13.33%), and sinus arrest, vertigo, vision blurred, fatigue, gait disturbance, pyrexia, hordeolum, rhinitis, chronic sinusitis, fall, dehydration, spinal column ste, headache, pollakiuria, and asthma (1/15 or 6.67%) were reported in group 1. Cardiac failure congestive, hematuria (3/15 or 20.00%), arteriosclerosis, squamous cell carcinoma, dyspnoea (2/15 or 13.33%), eye pruritus, eye swelling, dysphagia, nausea, chest pain, urinary tract infection, prostatic specific antigen, gout, inguinal mass, pain in extremity, prostatitis, breast mass, cough, epistaxis, alopecia, stasis dermatitis, cardioversion, implantable defibrillator replacement, and tooth extraction (1/15 or 13.33%) were observed in group 2. The results of this study showed the effectiveness of both dosages of cells on reduction of scar size. However, only the 100 million dosages enhanced ejection fraction [112]. Investigation of MSC-therapy for treating osteoarthritis of the knee (NCT02958267) was performed on 32 participants in two groups of BMAC injection and PRP injection (*n* = 17) and Gel-One® hyaluronate injection (*n* = 15). This clinical trial report a mortality rate of zero for all groups and adverse effects of nausea and vomiting (1/17 or 5.88%) for the BMAC injection and PRP injection groups. No data is available about the success of this therapy.

The purpose of the clinical trial NCT02509156 was to examine the safety, feasibility, and therapeutic efficacy of allogeneic human-MSCs delivered through transendocardial injection to cancer survivors with left ventricular (LV) dysfunction secondary to anthracycline-induced cardiomyopathy (AIC). 37 subjects in two groups of Allo-MSCs (*n* = 20) and placebo (*n* = 17) were examined in terms of the adverse effect of this trial. A mortality rate of 5.00% and 0% was reported for Allo-MSCs and placebo groups. Serious adverse events including cardiac disorders, sudden cardiac death, procedural pneumothorax, hyperglycaemia, osteoarthritis, transient ischaemic attack, acute kidney injury, and hypotension were reported for the Allo-MSCs group with a total rate of 25.00%. Serious adverse events of cardiac, gastrointestinal, and hepatobiliary disorders, infections and infestations, fall, hyponatraemia, syncope, product issues (lead dislodgement and device lead damage), acute kidney injury, menorrhagia, and hypotension were reported for the placebo group with a total rate of 64.71%. The article published according to the results of this trial does not mention that this therapy is safe or not. The author of this trial stated that if the therapy will be safe and feasible, we will conduct a larger phase II/III trial to examine its therapeutic efficacy [113].

## 9. Future Prospective

The large number of trials focusing on MSCs therapy shows the importance of this therapy from point of view of scientists, and if these trials will be successful, they will change human life positively. Despite the increasing rate of development in MSCs therapy, it has not been commonly used by clinicians because of challenges such as the timing and optimum dosage of MSC administration. There are some conditions that have been treated in preclinical experiments with MSCs but are yet to be translated into clinical trials. Some of these disorders are epilepticus, glioblastoma, hypoxic-ischemic encephalopathy, loss of retinal ganglion cells, nerve regeneration, azoospermia, and nephron generation in kidney cortices (Table 4). Therefore, it is encouraged to study the possibility of clinical trials of MSC therapy for such disease in the future. The potential of MSCs brings to mind the idea that the medicine of tomorrow can treat some of the incurable diseases including those related to aging.

## Figures and Tables

**Table 1:** Completed clinical trials on mesenchymal stromal cell-based therapy during 2016-2020 (U. S. National Library of Medicine).

Organ system	Disease/syndrome	Phase	Date	Country	Source	Transplantation	CT code
Cardiovascular	Cardiomyopathy	1	2019	United States	ND	Allotransplant	NCT02509156
Cardiovascular	Graft versus host disease	1 and 2	2016	Pakistan	Bone marrow	Allotransplant	NCT02824653
Cardiovascular	Ischemic cardiomyopathy	2	2020	United States	Bone marrow	Autotransplant	NCT02501811
Gastrointestinal	Cirrhosis	4	2020	India	Bone marrow	Autotransplant	NCT04243681
Gastrointestinal	Xerostomia	2	2017	Denmark	Adipose tissue	Autotransplant	NCT02513238
Gastrointestinal	Type 2 diabetes mellitus	1 and 2	2019	Vietnam	Bone marrow	Autotransplant	NCT03343782
Immune	Discordant immunological response in HIV	1 and 2	2019	Spain	Adipose tissue	Allotransplant	NCT02290041
Immune	Systemic lupus erythematosus	1	2018	United States	Umbilical cord	Allotransplant	NCT03171194
Integumentary	Atopic dermatitis	1	2017	Korea	ND	Autotransplant	NCT02888704
Integumentary	Caesarean section scars	2	2018	China	Umbilical cord	Allotransplant	NCT02772289
Integumentary	Chronic ulcer	1	2020	Indonesia	Conditioned medium Wharton's jelly	Allotransplant	NCT04134676
Integumentary	Chronic venous ulcer	1 and 2	2020	Germany	Skin	Allotransplant	NCT03257098
Integumentary	Diabetic foot ulcer	2	2016	Korea	Adipose tissue	Allotransplant	NCT02619877
Integumentary	Diabetic neuropathic ulcer	1 and 2	2020	Germany	Skin	Allotransplant	NCT03267784
Integumentary	Gingival recession	1 and 2	2019	Belarus	Adipose tissue	Autotransplant	NCT04434794
Integumentary	Perianal fistula	1	2019	United States	ND	Autotransplant	NCT02589119
Integumentary	Recessive dystrophic epidermolysis bullosa	1 and 2	2020	Korea	Umbilical cord	Allotransplant	NCT04520022
Integumentary	Rectovaginal fistula	1	2019	Russia	Adipose tissue	Autotransplant	NCT03643614
Integumentary	Skin scar	1 and 2	2019	Poland	Adipose tissue	Autotransplant	NCT03887208
Integumentary	Skin wound	1	2019	Pakistan	Umbilical cord	Allotransplant	NCT04219657
Integumentary	Surgical leak fistula	1	2019	United States	Adipose tissue	Autotransplant	NCT02807389
Integumentary	Ultrafiltration failure	1 and 2	2017	Iran	Adipose tissue	Allotransplant	NCT02801890
Nervous	Alzheimer's disease	1 and2	2019	United States	Adipose tissue	Autotransplant	NCT03117738
Nervous	Brain death	1	2020	India	ND	ND	NCT02742857
Nervous	Cerebral infarction	1 and 2	2017	Korea	Umbilical cord	Allotransplant	NCT02378974
Nervous	Corneal ulcer Corneal disease Corneal dystrophy	1&2	2019	Belarus	Adipose tissue	Autotransplant	NCT04484402
Nervous	Motor neuron disease	1	2016	Brazil	ND	Autotransplant	NCT02987413
Nervous	Multiple sclerosis	2	2019	Canada	ND	Autotransplant	NCT02239393
Nervous	Multiple sclerosis	1 and 2	2020	Jordan	Umbilical cord	Allotransplant	NCT03326505
Nervous	Multiple sclerosis	2	2018	Israel	Bone marrow	Autotransplant	NCT02166021
Nervous	Multiple sclerosis	1 and2	2017	Spain	Bone marrow	Autotransplant	NCT02495766
Nervous	Ocular corneal burn	2	2017	China	Bone marrow	ND	NCT02325843
Nervous	Parkinson's disease	1	2019	United States	Bone marrow	Autotransplant	NCT02611167
Nervous	Refractory epilepsy	1	2019	Russia	Adipose tissue	Autotransplant	NCT03676569
Nervous	Retinitis pigmentosa	3	2019	Turkey	Wharton's jelly	Allotransplant	NCT04224207
Nervous	Spinal cord injuries	1 and 2	2018	Jordan	Bone marrow Adipose tissue	Autotransplant	NCT02981576
Nervous	Spinal cord injuries	1 and 2	2020	China	Umbilical cord	Allotransplant	NCT02481440
Nervous	Spinal cord injuries	2	2017	Spain	Bone marrow	Autotransplant	NCT02570932
Nervous	Spinal cord injury	1 and 2	2019	Spain	Wharton's jelly	Allotransplant	NCT03003364
Reproductive	Atrophic endometrium	2	2019	Russia	Bone marrow	Autotransplant	NCT03166189
Reproductive	Erectile dysfunction	1	2018	Korea	Bone marrow	Autotransplant	NCT02344849
Reproductive	Erectile dysfunction	1	2018	Jordan	Wharton's jelly	Allotransplant	NCT02945449
Reproductive	Erectile dysfunction	1 and 2	2019	Jordan	Wharton's jelly	Allotransplant	NCT03751735
Reproductive	Fistula vagina	1	2020	United States	ND	Autotransplant	NCT03220243
Reproductive	Ovarian cancer	1	2019	United States	ND	Autotransplant	NCT02530047
Reproductive	Premature ovarian failure	1 and 2	2018	China	Umbilical cord	Allotransplant	NCT02644447
Respiratory	Acute respiratory distress syndrome	1	2019	United States	Bone marrow	Allotransplant	NCT02804945
Respiratory	Bronchopulmonary dysplasia	1 and 2	2016	United States	Umbilical cord	Allotransplant	NCT02381366
Respiratory	COVID-19 Acute respiratory distress syndrome	1 and 2	2020	United States	Umbilical cord	Allotransplant	NCT04355728
Respiratory	COVID-19 Prophylaxis	1	2020	United States	Umbilical cord	Allotransplant	NCT04573270
Respiratory	COVID-19	2	2020	China	Umbilical cord	Allotransplant	NCT04288102
Respiratory	COVID-19	1	2020	China	Exosome Adipose tissue	Allotransplant	NCT04276987
Respiratory	Laryngotracheal stenosis	1 and 2	2019	Belarus	Olfactory mucosa	Autotransplant	NCT03130374
Respiratory	Noncystic fibrosis bronchiectasis	1	2019	United States	Bone marrow	Allotransplant	NCT02625246
Respiratory	Pneumoconiosis	1	2019	China	Umbilical cord	Allotransplant	NCT02668068
Skeletal	Bone fracture	1 and 2	2020	India	Adipose tissue	Autotransplant	NCT04340284
Skeletal	Dental implant therapy	1 and 2	2017	Greece	Bone marrow	Autotransplant	NCT03070275
Skeletal	Knee osteoarthritis	2	2018	United States	Bone marrow	Autotransplant	NCT02958267
Skeletal	Knee osteoarthritis	1 and 2	2018	Canada	Bone marrow	Autotransplant	NCT02351011
Skeletal	Knee osteoarthritis	2	2018	United States	Adipose tissue	Autotransplant	NCT02674399
Skeletal	Osteoarthritis	1	2018	China	Adipose tissue	Allotransplant	NCT02641860
Skeletal	Osteonecrosis	1 and 2	2018	Spain	Bone marrow	Autotransplant	NCT01605383
Skeletal	Osteoporosis Spinal fractures	1	2016	Spain	Bone marrow	Autotransplant	NCT02566655
Skeletal	Rheumatoid arthritis	1	2018	Iran	Bone marrow	Autotransplant	NCT03333681
Skeletal	Rheumatoid arthritis	1 and 2	2020	United States	Adipose tissue	Autotransplant	NCT03691909
Urinary	Stress urinary incontinence	1 and 2	2019	Belarus	Adipose tissue	Autotransplant	NCT04446884
Urinary	Stress urinary incontinence	3	2016	Egypt	Bone marrow	Autotransplant	NCT02334878

ND: no data.

**Table 2:** Active clinical trials on mesenchymal stromal/stem cell-based therapy started during 2016-2020 (U. S. National Library of Medicine).

Organ system	Disease/syndrome	Phase	Date	Country	Source	Transplantation	CT code
Cardiovascular	Hypoplastic left heart syndrome	1 and 2	2018	United States	Bone marrow	Allotransplant	NCT03525418
Gastrointestinal	Cystic fibrosis	1	2016	United States	Bone marrow	Allotransplant	NCT02866721
Gastrointestinal	Diabetes	1 and 2	2017	United States	ND	Allotransplant	NCT02886884
Gastrointestinal	Inflammatory bowel	1 and 2	2017	Jordan	Wharton's jelly	Allotransplant	NCT03299413
Gastrointestinal	Primary sclerosing cholangitis	1 and 2	2017	China	Umbilical cord	Allotransplant	NCT03516006
Gastrointestinal	Xerostomia	1	2019	Denmark	Adipose tissue	Allotransplant	NCT03874572
Immune	Graft versus host disease	1 and 2	2016	Spain	Adipose tissue	Autotransplant	NCT02687646
Immune	Graft versus host disease	1	2018	United States	Umbilical cord Wharton's jelly	Allotransplant	NCT03158896
Integumentary	Epidermolysis bullosa	1 and 2	2018	Spain	Bone marrow	Allotransplant	NCT04153630
Integumentary	Epidermolysis bullosa	1 and 2	2019	United Kingdom	Skin	Allotransplant	NCT03529877
Integumentary	Inflammation	1 and 2	2018	United States	Bone marrow	Allotransplant	NCT03059355
Integumentary	Psoriasis	1 and 2	2017	China	Adipose tissue	Allotransplant	NCT03265613
Nervous	Alcoholism	1 and 2	2018	United States	ND	Allotransplant	NCT03265808
Nervous	Alzheimer's disease	1 and 2	2020	United States	Adipose tissue	Autotransplant	NCT04228666
Nervous	Alzheimer's disease	1	2016	United States	Bone marrow	Allotransplant	NCT02600130
Nervous	Amyotrophic lateral sclerosis	3	2017	United States	Umbilical cord	Allotransplant	NCT03280056
Nervous	Cerebral palsy	1 and 2	2016	Jordan	Bone marrow	Autotransplant	NCT03078621
Nervous	Cerebral palsy	2	2017	Iran	Umbilical cord	Allotransplant	NCT03795974
Nervous	Cerebral palsy	1 and 2	2018	United States	Umbilical cord	Allotransplant	NCT03473301
Nervous	Huntington disease	2	2018	Brazil	Dental pulp	Allotransplant	NCT03252535
Nervous	Huntington disease	1	2017	Brazil	Dental pulp	Allotransplant	NCT02728115
Nervous	Multiple sclerosis	2	2018	United States	Bone marrow	Autotransplant	NCT03355365
Nervous	Multiple sclerosis	2	2019	United States	ND	Autotransplant	NCT03799718
Nervous	Multiple system atrophy	1	2018	Korea	Bone marrow	Autotransplant	NCT04495582
Nervous	Nervous injury	1 and 2	2017	China	Umbilical cord	Allotransplant	NCT03291366
Nervous	Parkinson's disease	2	2017	Belarus	Bone marrow	Autotransplant	NCT04146519
Nervous	Spinal cord injury	1	2017	United States	Adipose tissue	Autotransplant	NCT03308565
Respiratory	COVID-19	1 and 2	2020	Spain	Adipose tissue	Allotransplant	NCT04366323
Respiratory	COVID-19	2	2020	United States	Adipose tissue	Allotransplant	NCT04362189
Respiratory	COVID-19	2	2020	United States	Adipose tissue	Autotransplant	NCT04349631
Respiratory	COVID-19	1 and 2	2020	France	Umbilical cord Wharton's jelly	Allotransplant	NCT04333368
Skeletal	Knee osteoarthritis	1	2019	United States	Adipose tissue	Autotransplant	NCT04043819
Skeletal	Lateral epicondylitis	2	2018	Korea	Adipose tissue	Allotransplant	NCT03449082
Skeletal	Osteoarthritis	2	2017	Korea	Adipose tissue	Autotransplant	NCT03509025
Skeletal	Osteoarthritis	3	2019	Korea	Adipose tissue	Autotransplant	NCT03990805
Skeletal	Osteoarthritis	1	2016	Jordan	Bone marrow	Autotransplant	NCT03067870
Urinary	Fistula	1	2017	United States	Adipose tissue	Autotransplant	NCT02808208
Urinary	Kidney injury	1 and 2	2017	United States	Bone marrow	Allotransplant	NCT03015623
Urinary	Nephrosis	2	2016	China	Bone marrow	Autotransplant	NCT02966717

ND: no data.

**Table 3:** Recruiting clinical trials on mesenchymal stromal/stem cell-based therapy started during 2016-2020 (U. S. National Library of Medicine).

Organ system	Disease/syndrome	Phase	Date	Country	Source	Transplantation	CT code
Cardiovascular	Abdominal aortic aneurysm	1	2016	United States	ND	Allotransplant	NCT02846883
Cardiovascular	Acute myocardial infarction	1 and 2	2019	Indonesia	Umbilical cord	Allotransplant	NCT04340609
Cardiovascular	Acute myocardial infarction	1	2019	Taiwan	Umbilical cord	Allotransplant	NCT04056819
Cardiovascular	Cardiomyopathy	1	2020	United States	Bone marrow	Allotransplant	NCT02962661
Cardiovascular	Cerebrovascular disorders	1 and 2	2019	Iran	Exosome	Allotransplant	NCT03384433
Cardiovascular	Critical limb ischemia	1	2020	Korea	Adipose tissue	Allotransplant	NCT04661644
Cardiovascular	Critical limb ischemia	2	2020	France	Adipose tissue	Autotransplant	NCT03968198
Cardiovascular	Diabetic foot ulcer	3	2020	Korea	Adipose tissue	Allotransplant	NCT04569409
Cardiovascular	Diabetic foot ulcer	1	2020	China	Umbilical cord	Allotransplant	NCT04464213
Cardiovascular	Heart defects	1 and 2	2017	United States	ND	Allotransplant	NCT03079401
Cardiovascular	Heart failure	1	2016	United States	Bone marrow	Allotransplant	NCT02408432
Cardiovascular	Heart failure	2 and 3	2018	Poland	Wharton's jelly	Allotransplant	NCT03418233
Cardiovascular	Ischemia	1	2017	United States	Bone marrow	Allotransplant	NCT02685098
Cardiovascular	Ischemia reperfusion injury	2	2020	United States	Adipose tissue	Allotransplant	NCT04388761
Cardiovascular	Ischemic heart disease	1 and 2	2018	China	Bone marrow	Autotransplant	NCT03397095
Cardiovascular	Ischemic stroke	1	2020	Taiwan	Umbilical cord	Allotransplant	NCT04434768
Cardiovascular	Ischemic stroke	1	2019	Taiwan	Umbilical cord	Allotransplant	NCT04097652
Cardiovascular	Myocardial infarction	1	2019	Spain	Umbilical cord	Allotransplant	NCT03798353
Cardiovascular	Myocardial infarction	2 and 3	2017	Poland	Wharton's jelly	Allotransplant	NCT03404063
Cardiovascular	Myocardial ischemia	2	2016	France	Bone marrow	Autotransplant	NCT02462330
Digestive	Crohn colitis	1 and 2	2020	United States	Bone marrow	Allotransplant	NCT04548583
Digestive	Crohn disease	1	2018	United States	ND	Autotransplant	NCT03449069
Digestive	Crohn disease	1 and 2	2018	Belgium	ND	ND	NCT03901235
Digestive	Fistula	1 and 2	2020	United States	Bone marrow	Allotransplant	NCT04519671
Digestive	Fistula	1 and 2	2020	United States	Bone marrow	Allotransplant	NCT04519684
Digestive	Hepatitis B	1	2018	China	Umbilical cord	Allotransplant	NCT03826433
Digestive	Liver cirrhosis	1 and 2	2018	Singapore	Bone marrow	Autotransplant	NCT03626090
Digestive	Liver cirrhosis	2	2019	China	Umbilical cord	Allotransplant	NCT03945487
Digestive	Liver cirrhosis	1 and 2	2018	Indonesia	Umbilical cord	Allotransplant	NCT04357600
Digestive	Liver failure	1 and 2	2019	Germany	Skin	Allotransplant	NCT03860155
Digestive	Liver transplantation	1	2017	Germany	Bone marrow	Allotransplant	NCT02957552
Digestive	Primary biliary cirrhosis	1 and 2	2019	Vietnam	Umbilical cord	Allotransplant	NCT04522869
Digestive	Rectovaginal fistula	1 and 2	2020	United States	Bone marrow	Allotransplant	NCT04519697
Digestive	Ulcerative colitis	1 and 2	2018	China	Adipose tissue	Allotransplant	NCT03609905
Digestive	Ulcerative colitis	1	2020	United States	Adipose tissue	Autotransplant	NCT04312113
Digestive	Ulcerative colitis	1 and 2	2020	United States	Bone marrow	Allotransplant	NCT04543994
Endocrine	Diabetic kidney disease	1	2019	United States	Adipose tissue	Autotransplant	NCT03840343
Endocrine	Type 1 diabetes	1	2017	Jordan	Adipose tissue	Allotransplant	NCT02940418
Endocrine	Type 1 diabetes	1 and 2	2019	Sweden	Wharton's jelly	Allotransplant	NCT03973827
Endocrine	Type 2 diabetes	1 and 2	2016	Indonesia	Bone marrow Umbilical cord	Autotransplant Allotransplant	NCT04501341
Endocrine	Type 2 diabetes	1 and 2	2020	China	Umbilical cord	Allotransplant	NCT04441658
Immune	Acute graft versus host disease	1 and 2	2019	Malaysia	Umbilical cord	Allotransplant	NCT03847844
Immune	Diabetic foot ulcer	1	2019	United States	Umbilical cord	Allotransplant	NCT04104451
Immune	Drug resistant	1 and 2	2020	China	Adipose tissue	Allotransplant	NCT04544215
Immune	Keratoconjunctivitis sicca	2	2020	Denmark	Adipose tissue	Allotransplant	NCT04615455
Immune	Lupus erythematosus	1 and 2	2019	Belarus	Olfactory mucosa	Allotransplant	NCT04184258
Immune	Lymphoblastic leukemia	2	2017	United States	Umbilical cord	Autotransplant	NCT03096782
Immune	Primary sclerosing cholangitis Autoimmune hepatitis	1 and 2	2018	United Kingdom	Umbilical cord	Allotransplant	NCT02997878
Immune	Renal lupus	2	2019	Chile	Umbilical cord	Allotransplant	NCT03917797
Immune	Type 1 diabetes	1	2020	United States	Umbilical cord	Allotransplant	NCT04061746
Immune	Type 1 diabetes	1 and 2	2017	Sweden	Wharton's jelly	Allotransplant	NCT03406585
Integumentary	Caesarean section scars	2	2020	China	Perinatal tissue	Allotransplant	NCT04034615
Integumentary	Psoriasis	1 and 2	2019	China	Adipose tissue	Allotransplant	NCT03392311
Integumentary	Wound	1	2019	China	ND Conditioned medium	ND	NCT04235296
Nervous	Alzheimer's disease	1 and 2	2020	China	Adipose tissue	Allotransplant	NCT04388982
Nervous	Alzheimer's disease	1	2019	United States	Umbilical cord	Allotransplant	NCT04040348
Nervous	Amyotrophic lateral sclerosis	2	2017	United States	Adipose tissue	Autotransplant	NCT03268603
Nervous	Amyotrophic lateral sclerosis	1 and 2	2020	Poland	Wharton's jelly	Allotransplant	NCT04651855
Nervous	Cerebral palsy	1 and 2	2020	Indonesia	Umbilical cord	Allotransplant	NCT04314687
Nervous	Cornea	1	2020	United States	Bone marrow	Allotransplant	NCT04626583
Nervous	Dry eye	1 and 2	2020	China	Umbilical cord Exosome	Allotransplant	NCT04213248
Nervous	Nonarteritic anterior ischemic optic neuropathy	2	2018	Spain	Bone marrow	Allotransplant	NCT03173638
Nervous	Parkinson's disease	1 and 2	2018	Jordan	Umbilical cord	Allotransplant	NCT03684122
Nervous	Parkinson's disease	2	2020	United States	Bone marrow	Allotransplant	NCT04506073
Nervous	Recurrent glioblastoma	1	2019	United States	Bone marrow	Allotransplant	NCT03896568
Nervous	Sclerosis	1	2016	Jordan	Bone marrow	Autotransplant	NCT03069170
Nervous	Spinal cord injuries	1 and 2	2016	Spain	Adipose tissue	Allotransplant	NCT02917291
Nervous	Spinal cord injuries	2	2020	United States	Adipose tissue	Autotransplant	NCT04520373
Nervous	Spinal cord injuries	1	2017	Jordan	Bone marrow	Autotransplant	NCT04288934
Nervous	Spinal cord injuries	2	2019	China	Umbilical cord	Allotransplant	NCT03521336
Nervous	Spinal cord injuries	2	2019	China	Umbilical cord	Allotransplant	NCT03521323
Nervous	Spinal cord injuries	2	2019	China	Umbilical cord	Allotransplant	NCT03505034
Nervous	Stroke	1 and 2	2020	Netherlands	Bone marrow	Allotransplant	NCT03356821
Nervous	Traumatic brain injury	1 and 2	2020	United States	Adipose tissue	Autotransplant	NCT04063215
Reproductive	Erectile dysfunction	2	2020	Korea	Bone marrow	Autotransplant	NCT04594850
Reproductive	Intraventricular hemorrhage	2	2017	Korea	Umbilical cord	Allotransplant	NCT02890953
Reproductive	Uterine scar	1	2020	China	Umbilical cord	Allotransplant	NCT03181087
Reproductive	Uterine scar	2	2020	China	Umbilical cord	Allotransplant	NCT02968459
Reproductive	Uterus injury	2	2020	China	Umbilical cord	Allotransplant	NCT03386708
Respiratory	Acute respiratory distress syndrome	1 and 2	2019	Spain	Adipose tissue	Allotransplant	NCT04289194
Respiratory	Acute respiratory distress syndrome	2	2019	United States	Bone marrow	Allotransplant	NCT03818854
Respiratory	Acute respiratory distress syndrome	1 and 2	2020	United States	ND	Allotransplant	NCT04524962
Respiratory	Acute respiratory distress syndrome	1 and 2	2019	United Kingdom	Umbilical cord	Allotransplant	NCT03042143
Respiratory	Adenocarcinoma of lung	1 and 2	2019	United Kingdom	ND	Allotransplant	NCT03298763
Respiratory	Bronchopulmonary dysplasia	1	2019	United States	Bone marrow Exosome	Allotransplant	NCT03857841
Respiratory	Bronchopulmonary dysplasia	1	2019	Spain	ND	ND	NCT02443961
Respiratory	Bronchopulmonary dysplasia	1 and 2	2019	China	Umbilical cord	Allotransplant	NCT03645525
Respiratory	Bronchopulmonary dysplasia	1	2018	China	Umbilical cord	Allotransplant	NCT03873506
Respiratory	Bronchopulmonary dysplasia	1 and 2	2019	China	Umbilical cord	Allotransplant	NCT03774537
Respiratory	Bronchopulmonary dysplasia	1	2018	China	Umbilical cord	Allotransplant	NCT03558334
Respiratory	Bronchopulmonary dysplasia	2	2018	Korea	Umbilical cord	Allotransplant	NCT03392467
Respiratory	Bronchopulmonary dysplasia	2	2019	Korea	Umbilical cord	Allotransplant	NCT04003857
Respiratory	Bronchopulmonary dysplasia	1	2018	Taiwan	Umbilical cord	Allotransplant	NCT03631420
Respiratory	Bronchopulmonary dysplasia	1	2019	Vietnam	Umbilical cord	Allotransplant	NCT04062136
Respiratory	Chronic lung allograft dysfunction	2	2017	Australia	Bone marrow	Allotransplant	NCT02709343
Respiratory	Chronic obstructive pulmonary disease	1	2020	United States	ND	ND	NCT04047810
Respiratory	Chronic obstructive pulmonary disease	1 and 2	2020	Vietnam	Umbilical cord	Allotransplant	NCT04433104
Respiratory	Chronic obstructive pulmonary disease	1	2020	Taiwan	Umbilical cord	Allotransplant	NCT04206007
Respiratory	COVID-19	1	2020	Mexico	Adipose tissue	Allotransplant	NCT04611256
Respiratory	COVID-19	1 and 2	2020	Belgium	Bone marrow	Allotransplant	NCT04445454
Respiratory	COVID-19	1	2020	Canada	Bone marrow	Allotransplant	NCT04400032
Respiratory	COVID-19	2	2020	Pakistan	Bone marrow	Allotransplant	NCT04444271
Respiratory	COVID-19	2	2020	Spain	Bone marrow	Allotransplant	NCT04361942
Respiratory	COVID-19	1	2020	Sweden	Bone marrow	Allotransplant	NCT04447833
Respiratory	COVID-19	1	2020	United States	Bone marrow	Allotransplant	NCT04397796
Respiratory	COVID-19	3	2020	United States	Bone marrow	Allotransplant	NCT04371393
Respiratory	COVID-19	1	2020	United States	Bone marrow	Allotransplant	NCT04629105
Respiratory	COVID-19	1	2020	United States	Cord blood	Allotransplant	NCT04565665
Respiratory	COVID-19	1 and 2	2020	United States	Wharton's jelly	Allotransplant	NCT04399889
Respiratory	COVID-19	1 and 2	2020	China	Dental pulp	Allotransplant	NCT04336254
Respiratory	COVID-19	1	2020	Indonesia	ND	Allotransplant	NCT04535856
Respiratory	COVID-19	2	2020	Mexico	ND	Allotransplant	NCT04416139
Respiratory	COVID-19	2	2020	Spain	ND	Allotransplant	NCT04615429
Respiratory	COVID-19	2	2020	United States	ND	Allotransplant	NCT04466098
Respiratory	COVID-19	1	2020	Brazil	ND	Allotransplant	NCT04525378
Respiratory	COVID-19	2 and 3	2020	Iran	ND Exosome	ND	NCT04366063
Respiratory	COVID-19	1 and 2	2020	United States	ND	Allotransplant	NCT04524962
Respiratory	COVID-19	1 and 2	2020	Ukraine	Umbilical cord	Allotransplant	NCT04461925
Respiratory	COVID-19	2	2020	Germany Israel	Umbilical cord	Allotransplant	NCT04614025
Respiratory	COVID-19	2	2020	United States	Umbilical cord	Allotransplant	NCT04389450
Respiratory	COVID-19	1 and 2	2020	China	Umbilical cord	Allotransplant	NCT04339660
Respiratory	COVID-19	1	2020	Indonesia	Umbilical cord	Allotransplant	NCT04457609
Respiratory	COVID-19	2	2020	Spain	Umbilical cord	Allotransplant	NCT04366271
Respiratory	COVID-19	1 and 2	2020	United States	Umbilical cord	Allotransplant	NCT04494386
Respiratory	COVID-19	2	2020	Pakistan	Umbilical cord	Allotransplant	NCT04437823
Respiratory	COVID-19	1	2020	China	Umbilical cord	Allotransplant	NCT04252118
Respiratory	COVID-19	1 and 2	2020	Turkey	Umbilical cord	Allotransplant	NCT04392778
Respiratory	COVID-19	1	2020	Jordan	Wharton's jelly	Allotransplant	NCT04313322
Respiratory	COVID-19	1 and 2	2020	Spain	Wharton's jelly	Allotransplant	NCT04390139
Respiratory	Healthy	1	2020	China	Adipose tissue exosome	Allotransplant	NCT04313647
Respiratory	Hoarseness	1 and 2	2020	Sweden	Bone marrow	Autotransplant	NCT04290182
Respiratory	Lung disease	1	2019	United States	Bone marrow	Allotransplant	NCT03929120
Respiratory	Pneumonia	2	2020	China	Umbilical cord	Allotransplant	NCT04269525
Respiratory	Pulmonary hypertension	1 and 2	2019	China	Adipose tissue	Autotransplant	NCT04055415
Skeletal	Alveolar bone atrophy	3	2020	Spain	Bone marrow	Autotransplant	NCT04297813
Skeletal	Aneurysmal bone cyst	1 and 2	2018	Jordan	Bone marrow	Autotransplant	NCT03066245
Skeletal	Cartilage defect	1	2018	United States	Adipose tissue	Autotransplant Allotransplant	NCT03672825
Skeletal	Disc disease	2	2017	Indonesia	Bone marrow	Allotransplant	NCT04499105
Skeletal	Disc disease	1	2019	China	Umbilical cord	Allotransplant	NCT04104412
Skeletal	Knee cartilage injury	1 and 2	2018	China	Adipose tissue	Autotransplant	NCT03955497
Skeletal	Knee osteoarthritis	2	2020	China	Adipose tissue	Allotransplant	NCT04208646
Skeletal	Knee osteoarthritis	1 and 2	2019	Taiwan	Adipose tissue	Allotransplant	NCT03943576
Skeletal	Knee osteoarthritis	1 and 2	2019	Poland	Adipose tissue	Autotransplant	NCT03869229
Skeletal	Knee osteoarthritis	1	2017	Jordan	Adipose tissue	Autotransplant	NCT02966951
Skeletal	Knee osteoarthritis	1 and 2	2020	Ukraine	Bone marrow	Allotransplant	NCT04453111
Skeletal	Knee osteoarthritis	1 and 2	2019	Korea	Bone marrow	Allotransplant	NCT04240873
Skeletal	Knee osteoarthritis	1	2018	United States	Bone marrow	Autotransplant	NCT03477942
Skeletal	Knee osteoarthritis	1	2019	Chile	Umbilical cord	Allotransplant	NCT03810521
Skeletal	Knee osteoarthritis	1	2019	Korea	Umbilical cord	Allotransplant	NCT04037345
Skeletal	Knee osteoarthritis	1	2020	Korea	Umbilical cord	Allotransplant	NCT04339504
Skeletal	Knee osteoarthritis	1	2017	Jordan	Wharton's jelly	Allotransplant	NCT02963727
Skeletal	Low back pain	1	2020	United States	Bone marrow	Allotransplant	NCT04410731
Skeletal	Meniscus injuries	2	2019	United States	Adipose tissue	Autotransplant	NCT04274543
Skeletal	Nonunion fracture	3	2017	France Germany	Bone marrow	Autotransplant	NCT03325504
Skeletal	Osteoarthritis	2	2016	France	Adipose tissue	Autotransplant	NCT02838069
Skeletal	Osteoarthritis	1	2016	United States	Adipose tissue	Autotransplant	NCT02805855
Skeletal	Osteoarthritis	1	2018	United States	Adipose tissue	Autotransplant	NCT03608579
Skeletal	Osteoarthritis	3	2019	United States	Bone marrow Adipose tissue	Autotransplant	NCT03818737
Skeletal	Osteoarthritis	2	2020	China	Umbilical cord	Allotransplant	NCT03383081
Skeletal	Osteoarthritis	1 and 2	2020	Indonesia	Umbilical cord	Allotransplant	NCT04314661
Skeletal	Osteoarthritis	1 and 2	2019	Poland	Wharton's jelly	Allotransplant	NCT03866330
Skeletal	Osteochondral fracture of talus	3	2019	Chile	Umbilical cord	Allotransplant	NCT03905824
Skeletal	Osteogenesis imperfecta	1 and 2	2019	Sweden	Fetal liver	Allotransplant	NCT03706482
Skeletal	Osteoporosis	2	2020	Indonesia	Umbilical cord	Allotransplant	NCT04501354
Skeletal	Rheumatoid arthritis	1	2017	United States	ND	Allotransplant	NCT03186417
Skeletal	Rheumatoid arthritis	1 and 2	2018	Korea	Umbilical cord	Allotransplant	NCT03618784
Skeletal	Rotator cuff tear	2	2020	Brazil	ND	ND	NCT03362424
Skeletal	Spinal tuberculosis	2	2017	Indonesia	ND	ND	NCT04493918
Urinary	COVID-19	1 and 2	2020	United States	Bone marrow	Allotransplant	NCT04445220
Urinary	Kidney diseases	1 and 2	2019	Bangladesh	Adipose tissue	Autotransplant	NCT03939741
Urinary	Kidney diseases	1 and 2	2017	Ireland Italy United Kingdom	Bone marrow	Allotransplant	NCT02585622
Urinary	Nephropathy	1	2020	Japan	Adipose tissue	Allotransplant	NCT04342325
Urinary	Pelvic radiation	2	2019	France	ND	ND	NCT02814864
Urinary	Renal	1	2020	United States	Adipose tissue	Allotransplant	NCT04392206
Urinary	Renal transplantation	1	2018	United States	ND	Allotransplant	NCT03504241
Urinary	Renal transplantation	2	2016	United States	ND	Autotransplant	NCT03478215
Urinary	Type 2 diabetes	1 and 2	2020	China	Umbilical cord	Allotransplant	NCT04216849

ND: no data.

**Table 4:** Diseases can be tested clinically for treatment with mesenchymal stromal/stem cells in human based on the successful *in vivo* model studies.

Organ system	Disease/syndrome	Source	Transplantation	References
Cardiovascular	Myelodysplastic syndromes	Bone marrow	Allotransplant	[114]
Gastrointestinal	Hepatic failure	Bone marrow	Allotransplant	[115]
Nervous	Epilepticus	Exosome	Allotransplant	[117]
Nervous	Glioblastoma	Bone marrow	Allotransplant	[118]
Nervous	Glioblastoma	Adipose tissue	Allotransplant	[119]
Nervous	Hypoxic-ischemic encephalopathy	Umbilical cord	Allotransplant	[120]
Nervous	Loss of retinal ganglion cells	Bone marrow-derived exosomes	Allotransplant	[121]
Nervous	Nerve regeneration	Bone marrow	Allotransplant	[122]
Reproductive	Azoospermia	Adipose tissue	Allotransplant	[123]
Reproductive	Azoospermia	Bone marrow	Allotransplant	[124]
Reproductive	Mammary adenocarcinoma	Adipose tissue	Allotransplant	[116]
Skeletal	Bone formation	ND	Allotransplant	[125]
Skeletal	Calvarial defects	Bone marrow Adipose tissue	Allotransplant	[126]
Skeletal	Periodontal defects	Exosome	Allotransplant	[127]
Urinary	Focal segmental glomerulosclerosis	Bone marrow	Allotransplant	[128]
Urinary	Nephron generation in kidney cortices	ND	Allotransplant	[129]

ND: no data.

## Data Availability

The data used to support the findings of this study are included within the article.
